# Acute hypoglycemia and risk of cardiac arrhythmias in insulin-treated type 2 diabetes and controls

**DOI:** 10.1530/EJE-21-0232

**Published:** 2021-06-03

**Authors:** Andreas Andersen, Jonatan I Bagger, Maria P A Baldassarre, Mikkel B Christensen, Kirsten U Abelin, Jens Faber, Ulrik Pedersen-Bjergaard, Jens J Holst, Tommi B Lindhardt, Gunnar Gislason, Filip K Knop, Tina Vilsbøll

**Affiliations:** 1 Steno Diabetes Center Copenhagen, Gentofte Hospital, Hellerup, Denmark; 2 Center for Clinical Metabolic Research, Herlev and Gentofte Hospital, University of Copenhagen, Hellerup, Denmark; 3 Department of Medicine and Aging Sciences, G. d’Annunzio University, Chieti, Italy; 4 Department of Clinical Pharmacology, Bispebjerg Hospital, University of Copenhagen, Copenhagen, Denmark; 5 Department of Medicine, Herlev and Gentofte Hospital, University of Copenhagen, Hellerup, Denmark; 6 Department of Clinical Medicine, Faculty of Health and Medical Sciences, University of Copenhagen, Copenhagen, Denmark; 7 Department of Endocrinology and Nephrology, Nordsjællands Hospital Hillerød, University of Copenhagen, Hillerød, Denmark; 8 Department of Biomedical Sciences, Faculty of Health and Medical Sciences, University of Copenhagen, Copenhagen, Denmark; 9 Novo Nordisk Foundation Center for Basic Metabolic Research, Faculty of Health and Medical Sciences, University of Copenhagen, Copenhagen, Denmark; 10 Department of Cardiology, Herlev and Gentofte Hospital, University of Copenhagen, Hellerup, Denmark; 11 The Danish Heart Foundation, Copenhagen, Denmark

## Abstract

**Objective:**

Hypoglycemia is associated with an increased risk of cardiovascular disease including cardiac arrhythmias. We investigated the effect of hypoglycemia in the setting of acute glycemic fluctuations on cardiac rhythm and cardiac repolarization in insulin-treated patients with type 2 diabetes compared with matched controls without diabetes.

**Design:**

A non-randomized, mechanistic intervention study.

**Methods:**

Insulin-treated patients with type 2 diabetes (*n* = 21, age (mean ± s.d.): 62.8 ± 6.5 years, BMI: 29.0 ± 4.2 kg/m^2^, HbA1c: 6.8 ± 0.5% (51.0 ± 5.4 mmol/mol)) and matched controls (*n* = 21, age: 62.2 ± 8.3 years, BMI 29.2 ± 3.5 kg/m^2^, HbA1c: 5.3 ± 0.3% (34.3 ± 3.3 mmol/mol)) underwent a sequential hyperglycemic and hypoglycemic clamp with three steady-states of plasma glucose: (i) fasting plasma glucose, (ii) hyperglycemia (fasting plasma glucose +10 mmol/L) and (iii) hyperinsulinemic hypoglycemia (plasma glucose < 3.0 mmol/L). Participants underwent continuous ECG monitoring and blood samples for counterregulatory hormones and plasma potassium were obtained.

**Results:**

Both groups experienced progressively increasing heart rate corrected QT (Fridericia’s formula) interval prolongations during hypoglycemia ((∆mean (95% CI): 31 ms (16, 45) and 39 ms (24, 53) in the group of patients with type 2 diabetes and controls, respectively) with similar increases from baseline at the end of the hypoglycemic phase (*P* = 0.43). The incidence of ventricular premature beats increased significantly in both groups during hypoglycemia (*P* = 0.033 and *P* < 0.0001, respectively). One patient with type 2 diabetes developed atrial fibrillation during recovery from hypoglycemia.

**Conclusions:**

In insulin-treated patients with type 2 diabetes and controls without diabetes, hypoglycemia causes clinically significant and similar increases in cardiac repolarization that might increase vulnerability for serious cardiac arrhythmias and sudden cardiac death.

## Introduction

Patients with type 2 diabetes have an increased risk of cardiovascular disease and death (1). The cornerstone in the treatment of type 2 diabetes is lifestyle intervention, glucose-lowering therapy along with the treatment of traditional cardiovascular risk factors including hypertension and dyslipidemia. Several large-scale clinical trials that investigated the effect of strict glycemic control failed to demonstrate any cardiovascular risk reduction (2), and in the ACCORD trial, strict glycemic control resulted in a three-fold increase in severe hypoglycemia and excess mortality (3). The notion of an association between hypoglycemia and cardiovascular disease is supported by multiple studies; however, a causal relationship has not been proven (4).

In individuals with type 2 diabetes and high cardiovascular risk, current consensus recommends treatment with a sodium-glucose cotransporter 2 (SGLT2) inhibitor or a glucagon-like peptide 1 (GLP-1) receptor agonist with proven cardiovascular disease benefit and low risk of hypoglycemia (5). Nevertheless, insulin therapy is often necessary to achieve satisfactory glycemic control thereby increasing the risk of hypoglycemia.

Potential mechanisms by which hypoglycemia may promote cardiovascular disease include blood coagulation abnormalities, inflammation, endothelial dysfunction and hemodynamic changes (4). Additionally, the counterregulatory response during hypoglycemia with pronounced sympathoadrenal activation is suspected to cause abnormal cardiac repolarization and induce cardiac arrhythmias (6). It is well established that heart rate-corrected QT interval (QTc) is prolonged during hypoglycemia in patients with type 1 diabetes and type 2 diabetes (7). QTc prolongation is associated with an increased risk of ventricular arrhythmias and is a strong predictor of cardiovascular mortality in type 2 diabetes (8, 9), which provides a potential explanation for the increased mortality observed in the ACCORD trial (3). In insulin-treated patients with type 2 diabetes, spontaneous episodes of hypoglycemia are associated with an increased incidence of ventricular premature beats and nocturnal bradycardia (10).

In previous experimental studies of insulin-induced hypoglycemia, the effect of hypoglycemia on cardiac repolarization has been compared to cardiac repolarization during hyperinsulinemic euglycemia (11, 12, 13, 14). Hence, hypoglycemia was preceded by a relatively small decline in plasma glucose (PG). It has also been suggested that diabetes may aggravate the risk of cardiac rhythm abnormalities (11, 15); however, in the only study comparing the electrophysiological response to hypoglycemia in patients with type 2 diabetes with the response in controls without diabetes, only a fraction of the patients with type 2 diabetes were insulin-treated (11). Consequently, it is uncertain whether the results from this study translate into a risk population of insulin-treated patients.

In the present study, we aimed to investigate the effect of hypoglycemia in the setting of marked (but clinically relevant), acute glycemic fluctuations on cardiac rhythm, QTc interval and hormonal counterregulatory response in patients with insulin-treated type 2 diabetes compared to matched controls with normal glucose tolerance.

## Subjects and methods

### Approvals and registrations

The present study was carried out at Steno Diabetes Center Copenhagen and Center for Clinical Metabolic Research, Gentofte Hospital, Hellerup, Denmark from May 2017 to July 2019. The study was conducted in accordance with the Declaration of Helsinki as revised in 2008 and was approved by the Scientific Ethical Committee of the Capital Region of Denmark (ID No. H-16046212) and the Danish Data Protection Agency (ID No. HGH-2017-030) and registered at ClinicalTrials.gov (NCT03150030). Oral and written consent was obtained from all participants prior to inclusion in the study.

### Design and study population

The present study consisted of a combined hyperglycemic and hypoglycemic clamp. Twenty-one adults with insulin-treated type 2 diabetes with good glycemic control (HbA_1c_ ≤ 58 mmol/mol (7.5%)) and at least one microvascular complication were included. Complications to diabetes were defined as: peripheral neuropathy with vibration perception threshold of >25 mV determined by biothesiometry, moderate to severe retinopathy, and/or nephropathy (creatinine > 130 μmol/L and/or albuminuria). A group of 21 individuals with HbA_1c_ ≤ 42 mmol/mol (6.0%) and fasting PG (FPG) ≤ 6.1 mmol/L matched for sex, age and BMI served as controls. Individuals in the control group were excluded if they had any first-degree relatives with diabetes. Individuals with a medical history of episodes of arrhythmia, implantable cardiac defibrillator or pacemaker, severe heart failure (defined by the protocol as left ventricular ejection fraction < 25%) or structural heart disease (e.g. Wolf–Parkinson–White syndrome, congenital heart disease, severe valve disease) were excluded in both groups to ensure safety and avoid bias from any underlying arrhythmic substrate. Individuals with anemia or thyroid dysfunction (except well-regulated myxedema) were also excluded. Patients with type 2 diabetes were screened for hypoglycemia unawareness by asking the patients to which extent they were able to recognize episodes of hypoglycemia with answers categorized as (i) always (ii) sometimes or (iii) never. The latter two were categorized as impaired awareness.

### The clamp procedure

All participants were instructed to fast from 22:00 h and to avoid strenuous physical activity the day before the experimental day and not to take any medication (including insulin) on the experimental day. None of the participants measured plasma glucose levels < 3.0 mmol/L 24 h prior to the start of the experiment. A FPG of ≥ 4.0 and ≤ 12.0 mmol/L was required by protocol to initiate the experimental day. A peripheral intravascular catheter was placed in an anterior cubital vein of each forearm. One arm was heated throughout the clamp to facilitate repeated blood sampling and to achieve measures closer to arterial blood concentrations, and the intravascular catheter on the contralateral side was used for infusions. The clamp experiment was started between 8:30 h and 11:00 h. To avoid volume depletion due to fasting and blood drawing, an isotonic saline infusion was initiated at time 0 (infusion rate 200 mL/h) and kept at a constant infusion rate. Blood was drawn and analyzed for PG every 5 min. After 30 min of monitoring at FPG level (FPG phase), an individually adjusted infusion of 20% (weightt/volume) glucose was initiated and PG was increased to FPG + 10 mmol/L over a period of 50 min and kept constant for 30 min (hyperglycemic phase). At time 110 min, rapid acting insulin (Actrapid^®^, Novo Nordisk) was administrated as an i.v. bolus and an i.v. infusion was initiated. Insulin bolus dose was calculated as 9.1 IU times body surface area (m^2^) (BSA) estimated by the Mosteller formula (16) for patients with diabetes and 2.5 IU times BSA for controls. The corresponding insulin infusion rates were calculated as 20 IU/h times BSA for patients with diabetes and 5 IU/h times BSA for controls. If judged necessary by the principal investigator (per protocol), the insulin dose was halved in participants with diabetes and low daily insulin dose. A steady-state PG < 3.0 mmol/L was targeted to occur from 160 min to 190 min (hypoglycemic phase) followed by a spontaneous recovery period from 190 min to 220 min. The insulin infusion was discontinued after 10 min of PG < 3.0 mmol/L. If PG was < 2.2 mmol/L or the trend indicated that the next measurement would be below this level, PG was immediately corrected by glucose infusion.

### ECG monitoring and analysis

Participants underwent ECG monitoring with a three-lead (XYZ) Holter monitor (Spiderview version 3.03A, Microport CRM, Clamart, France). All three leads were bipolar with a positive (+) and a negative (−) component. The electrodes generating the X-, Y-, and Z-lead were placed in the right (−) and left mid-axillary line (+), the superior part of manubrium (−) and below xiphoid process (+), and at the fourth intercostal space left to manubrium(+) and posterior to fourth intercostal space (−), respectively. Signals were sampled at 1000 Hz and band filtered with a low pass 50 Hz filter. Pre-processing and data analysis were performed with SyneScope version 3.10, Microport CRM, Clamart, France. Holter recordings were manually reviewed for clinically relevant cardiac arhrythmias including atrial fibrillation, bradyarrhythmias (sinus arrest for more than 3 s, frequency below 30 b.p.m, or high grade atrioventricular (AV) block) and tachyarrhythmias (ventricular tachycardia). QTc interval was calculated as means within 10 min intervals from all three leads by an automated algorithm determining a mean complex waveform based on 30 s of ECG. The peak of the T-wave was determined by the parabola method and the end of the T-wave by the intersection between the maximum decreasing tangent and the isoelectric line (17). Heart rate correction was performed by both Fridericia’s correction (QTcF) and Bazett’s correction (QTcB). Mean heart rate was calculated for each phase in 1-min intervals. The software automatically detected supraventricular premature beats (prematurity threshold < 75%) and ventricular premature beats (prematurity threshold < 85%), and all events were subsequently manually validated.

### Blood samples and analyses

Blood samples for PG measurement were drawn into fluoride tubes and centrifuged immediately at 8200 ***g*** for 30 s before analyzed bedside using YSI model 2300 or 2900 biochemistry analyzer (Xylem Analytics, OH, USA). All other samples were drawn in lithium-heparin tubes (potassium), EDTA tubes (glucagon), tubes containing serum clot activator (cortisol and somatotropin), and iced serum tubes containing EGTA/glutathione preservation (noradrenaline). Plasma potassium was analyzed by the indirect ion-selective electrodes method (Atellica CH 930 Analyzer, Siemens Healthineers). Glucagon was analyzed by RIA, and cortisol and somatotropin were analyzed using a quantitative electrochemiluminescence assay (Cobas 8000, Roche Diagnostics). Noradrenalin was analyzed with an ELISA (ELISA) (Labor Diagnostika Nord, Nordhorn, Germany).

### Statistical analysis

Statistical analyses were performed with SAS studio version 3.71 (SAS Institute Inc., Cary, NC, USA). When describing baseline characteristics of the clamp procedure, data that followed an approximate normal distribution were presented as mean ± s.d., and skewed data were summarized as median (interquartile range). When describing endpoints, data were presented as mean and 95% CI or depicted as mean and s.e. in figures. To evaluate changes in QTc intervals, a general linear mixed model with phase as a repeated factor, ID as a random factor and an unrestricted covariance structure was applied. A similar general linear mixed model was applied to compare counts of ventricular and supraventricular premature beats during hyperglycemia and hypoglycemia with the FPG phase, and data were fitted to a negative binomial model to allow modeling of over-dispersed data and presented as incident rate ratios (IRR). Furthermore, a sensitivity analysis with the elimination of single participants with high count numbers demonstrated was performed. Hormonal counterregulatory response was measured as a baseline-subtracted area under the curve, with baseline defined as the end of the hyperglycemic phase. Differences in hormonal counterregulatory response were evaluated by an unpaired *t*-test. A *P*-value < 0.05 was considered statistically significant.

## Results

### Participants

The groups were well matched for age, sex and BMI (Table 1). Mean blood pressure was similar between groups, but antihypertensive therapy was more prevalent in the group of patients with type 2 diabetes. None of the participants had pre-existing ischemic heart disease or heart failure. Four participants in the group of patients with type 2 diabetes were unable to complete the full experimental day due to vasovagal symptoms (e.g. nausea) during the decline in PG before reaching hypoglycemia. One participant in the control group experienced a transient (2–3 min) seizure immediately after reaching hypoglycemia at a PG level of 2.4 mmol/L, but the seizure was immediately resolved when normal PG was restored. The participant was subsequently admitted for observation for 24 h before discharged without sequela. No explanation for the apparently low seizure threshold was found. All five participants, who were unable to complete the experimental day, were excluded from the analysis and replaced, resulting in 21 participants completing the experimental procedure in each group (Table 1).

**Table 1:** Baseline characteristics of the group of insulin-treated patients with type 2 diabetes and the group of controls matched according to age, sex and BMI. Binary data are presented as *n* (%) and continuous variables are presented as mean (s.d.) or median (interquartile range).

	Type 2 diabetes (*n* = 21)	Controls (*n* = 21)	*P*
Age (years)	62.8 (6.5)	62.2 (8.3)	0.805
Female	5 (24%)	5 (24%)	1.000
BMI (kg/m^2^)	29.0 (4.2)	29.2 (3.5)	0.873
HbA1c (mmol/mol)	51.0 (5.4)	34.3 (3.3)	<0.001
HbA1c (%)	6.8 (0.5)	5.3 (0.3)	<0.001
FPG (mmol/L)	7.2 (1.7)	5.7 (0.3)	<0.001
C-peptide (pmol/L)	259 (174–370)^a^	440 (344–573)	0.006
Insulin treatment	21 (100%)	NA	
Basal	12 (57%)		
Basal/bolus	4 (19%)		
Insulin mix	5 (24%)		
Daily insulin dose (IU)	38 (25–60)	NA	
Oral glucose-lowering drugs	16 (76%)	NA	
Metformin	15 (71%)		
SGLT2i	7 (33%)		
DDP-4i	4 (19%)		
Diabetes duration (years)	15.3 (6.6)	NA	
Impaired awareness	6 (29%)	NA	
Neuropathy	18 (86%)	NA	
Retinopathy	7 (33%)	NA	
Nephropathy	3 (14%)	NA	
Hypertension	18 (86%)	4 (19%)	<0.001
Beta-blockers	4 (19%)	1 (5%)	
Non-dihydropyridine calcium antagonist	1 (5%)	0 (%)	
Systolic blood pressure (mmHg)	138.0 (12.4)	137.0 (11.0)	0.794
Diastolic blood pressure (mmHg)	80.3 (7.5)	82.6 (7.3)	0.324
Heart rate (b.p.m)	67.1 (10.7)	60.0 (9.6)	0.027
Creatinine (µmol/L)	87.6 (25.2)	83.0 (10.3)	0.452
Potassium (mmol/L)	4.1 (0.4)	4.1 (0.3)	0.778

^a^Fasting levels while on active insulin treatment.

DDP-4i, dipeptidyl peptidase-4 inhibitor; FPG, fasting plasma glucose; SGLT2i, sodium-glucose transport protein 2 inhibitor; T2D, type 2 diabetes.

### The clamp procedure

**Figure 1:**
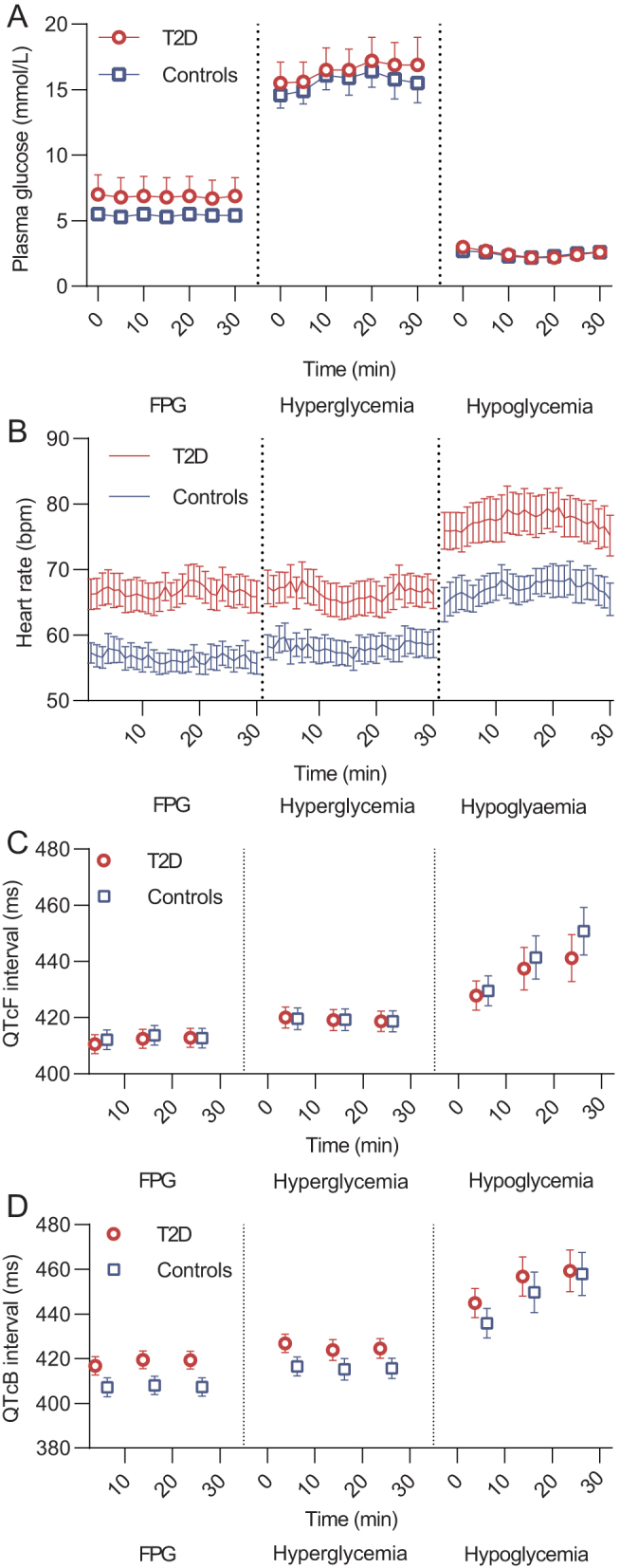
Plasma glucose, heart rate and QTc. (A) Plasma glucose (mean ± s.d., *n*  = 21 in both groups), (B) heart rate (mean ± s.e., *n*  = 21 (both groups)), (C) QTcF interval (mean ± s.e., *n*  = 21 (type 2 diabetes) and *n*  = 20 (controls)), (D) QTcB interval (mean ± s.e., *n*  = 21 (type 2 diabetes) and *n*  = 20 (controls)) during each steady-state of the experimental day in patients with type 2 diabetes (red circles) and controls (blue squares).FPG, fasting plasma glucose; T2D, type 2 diabetes; QTcB, heart rate-corrected QT interval by Bazett’s formula; QTcF, heart rate-corrected QT interval by Fridericia’s formula.

During the FPG phase and the hyperglycemic phase, PG levels were higher in the group of patients with type 2 diabetes compared with controls (6.9 ± 1.5 8 mmol/L vs 5.4 ± 0.5 8 mmol/L,* P* < 0.0001 and 16.4 ± 1.8 mmol/L vs 15.6 ± 1.3 mmol/L, *P* < 0.0001, respectively) (Fig. 1A

). Mean PG was similar between groups during hypoglycemia (2.5 ± 0.4 mmol/L in both groups, *P* = 0.53). At the end of the hyperglycemic phase, the cumulative amount of glucose infused was 53.0 (41.4–59.0) g and 62.8 (55.2–80.2) g in patients with type 2 diabetes and controls, respectively. Corresponding results at the end of the hypoglycemic phase were 53.6 (47.8–59.8) g and 69.9 (60.8–83.4) g, respectively. Time from ending the hyperglycemic phase until reaching PG ≤ 3.0 mmol/L exceeded the planned 50 min and was 72 ± 17 min and 59 ± 13 min in the group of patients with type 2 diabetes and controls, respectively. The PG decline rate within this period was 0.20 ± 0.04 mmol/L/min in the group of patients with type 2 diabetes and 0.22 ± 0.04 mmol/L/min in controls. Due to the delay in time to hypoglycemia, data on the recovery phase were not systematically obtained and are, therefore, not provided.

### Blood pressure and heart rate

Mean heart rate was higher in the group of patients with type 2 diabetes compared with controls during the FPG phase (Fig. 1B and Table 2). A marked increase in heart rate was observed in both groups during hypoglycemia. Systolic blood pressure remained unchanged in both groups when compared with the FPG phase, whereas diastolic blood pressure declined significantly during the hypoglycemic phase in both groups (Table 2).

**Table 2:** Changes in mean heart rate, blood pressure, plasma potassium and QTc interval during acute hyperglycemia and acute hypoglycemia compared with fasting plasma glucose in the group of insulin-treated patients with type 2 diabetes and the group of controls.

Parameter/group	FPG		Hyperglycemia			Hypoglycemia		
	Mean	95% CI	∆Mean	∆95% CI	*P*	∆Mean	∆95% CI	*P*
Heart rate (b.p.m)								
T2D	67	63; 71	−1	−2; 0	0.157	11	8; 14	<0.0001
Controls	56	52; 61	2	1; 3	0.002	11	8; 13	<0.0001
∆Groups			−2	−1, −4	0.002	0	−3, 4	0.835
Systolic BP (mmHg)								
T2D	133	125; 141	2	−4; 8	0.504	3	−4, 8	0.514
Controls	131	123; 139	6	0; 12	0.055	−6	−15; 3	0.199
∆Groups			−4	−13, 5	0.363	9	−4, 22	0.173
Diastolic BP (mmHg)								
T2D	78	73; 83	−1	−5; 2	0.456	−10	−16; −4	0.002
Controls	80	75; 85	1	−3; 5	0.613	−10	−16; −4	0.002
∆Groups			−2	−8, 3	0.377	0	−8, 9	0.946
Plasma potassium (mmol/L)								
T2D	4.1	4.0; 4.2	0.0	−0.1; 0.1	0.545	−1.0	−1.1; −0.9	<0.0001
Controls	4.1	4.0; 4.3	−0.2	−0.3; −0.1	0.002	−0.9	−1.0; −0.7	<0.0001
∆Groups			0.1	0.0, 0.3	0.074	−0.2	−0.3, 0.0	0.053
QTcF30 min (ms)								
T2D	410	404, 418	8	5, 11	<0.0001	31	16, 45	<0.0001
Controls	412	405, 419	7	3, 10	<0.001	39	24, 53	<0.0001
∆Groups			1	−3, 6	0.529	−8	−28, 12	0.434
QTcF_max_ (ms)								
T2D	447	432, 462	15	−6, 36	0.162	49	18, 79	<0.001
Controls	440	425, 455	3	−19, 25	0.790	55	24, 86	<0.001
∆Groups			12	−18, 43	0.426	−6	−50, 37	0.765
QTcB_30 min_ (ms)								
T2D	417	408, 425	8	3, 12	<0.001	42	27, 58	<0.0001
Controls	407	398, 416	8	4, 13	<0.001	51	35, 66	<0.0001
∆Groups			−1	−7, 5	0.799	−8	−30, 14	0.448
∆QTcB_max_ (ms)								
T2D	453	437, 470	16	−5, 37	0.133	60	30, 90	<0.001
Controls	449	432, 465	7	−14, 29	0.486	67	37, 97	<0.0001
∆Groups			8	−28, 32	0.574	−7	−50, 36	0.747

FPG, fasting plasma glucose; QTcB, heart rate-corrected QT by Bazett’s correction; QTcF, heart rate-corrected QT by Fridericia’s correction, ref, reference; T2D, type 2 diabetes.

**Figure 2:**
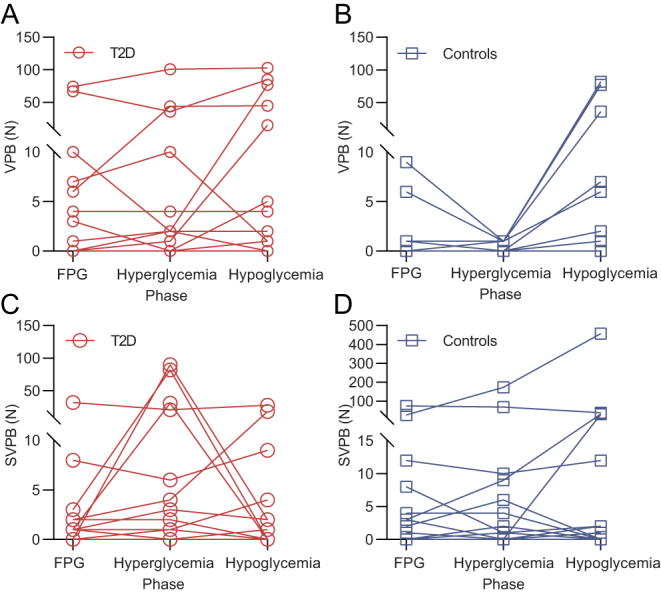
Ventricular and supraventricular premature beats. Individual counts of ventricular and supraventricular premature beats in patients with type 2 diabetes (A and C (red circles)) and controls (B and D (blue squares)). FPG, fasting plasma glucose; SVPB, supraventricular premature beats; T2D, type 2 diabetes; VPB, ventricular premature beats.

### Cardiac rhythm and QTc

None of the participants evolved any clinically significant arrhythmias during hypoglycemia. One participant with type 2 diabetes evolved asymptomatic atrial fibrillation immediately following the hypoglycemic phase before normal PG had been restored. The episode lasted for 5 h before spontaneously converting to sinus rhythm without any medical intervention. In one participant in the control group, QTc analysis was not possible due to technical issues. There was no difference in QTcF between the two groups during the FPG phase (Fig. 1C). QTcF increased progressively during hypoglycemia in both groups without any between group difference (Table 2). The increase in QTc was higher when applying Bazett’s correction. The maximal registered QTcF and QTcB increased significantly in both groups during hypoglycemia compared to the FPG phase without any between group difference (Table 2). When applying Bazett’s correction, maximal QTcB exceeded 500 ms in 48% of patients with type 2 diabetes and 55% of controls. During the hypoglycemic phase, the incidence of ventricular premature beats increased significantly in both groups when compared with the FPG phase (Fig. 2 and Table 3).

**Table 3:** Incident rate ratio of ventricular and supraventricular premature beats during hyperglycemia and hypoglycemia compared with fasting plasma glucose in the group of insulin-treated patients with type 2 diabetes and the group of controls.

Parameter/group	Hyperglycemia			Hypoglycemia		
	IRR	∆95% CI	*P*	IRR	∆95% CI	*P*
VPB						
T2D	1.2	(0.7; 2.1)	0.583	2.0	(1.1; 3.7)	0.033
Controls	0.2	(0.1; 0.4)	<0.0001	10.6	(3.4; 33.0)	<0.0001
SVPB						
T2D	4.6	(1.1; 19.3)	0.035	1.2	(0.7, 2.3)	0.511
Controls	2.0	(0.7; 5.7)	0.194	4.2	(0.9; 19.4)	0.065

FPG, fasting plasma glucose; IRR, incident rate ratio; SVPB, supraventricular premature beats; T2D, type 2 diabetes; VPB, ventricular premature beats.

### Counterregulatory hormones – glucagon, noradrenaline, cortisol and somatotropin

**Figure 3:**
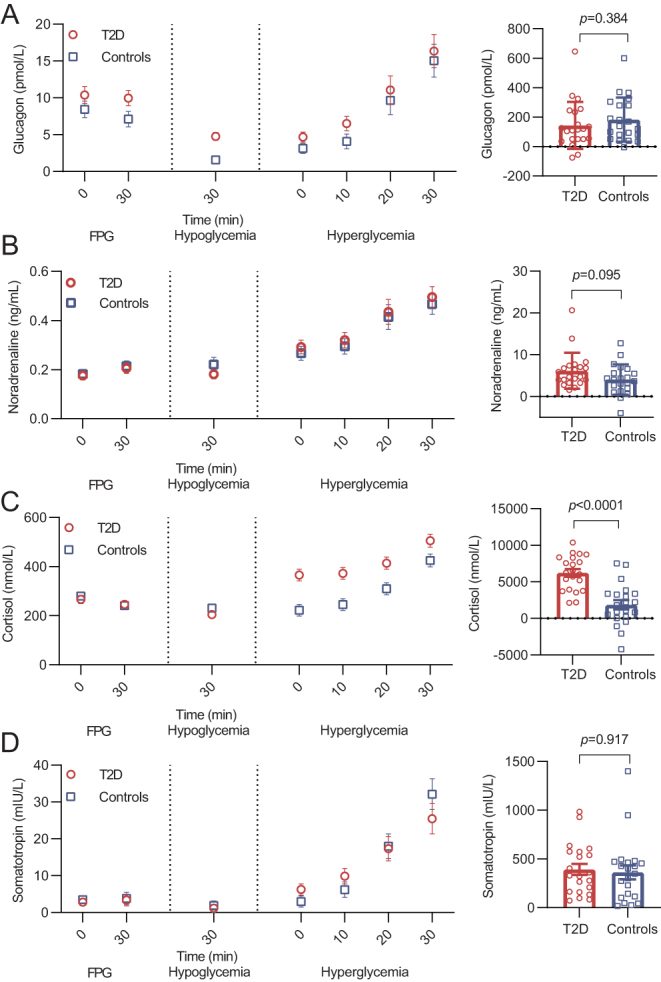
Hormonal counterregulatory response. Levels of (A) glucagon, (B) noradrenaline, (C) cortisol and (D) somatotropin during each steady-state phase of the experimental day in patients with type 2 diabetes (red circles) and controls (blue squares). On the left side, levels of the respective counterregulatory hormones during the clamp procedure are depicted (mean ± s.e., *n*  = 21 (both groups)). On the right side, hormonal counterregulatory response during the hypoglycemic phase is depicted as baseline subtracted area under the curve with the hyperglycemic phase applied as baseline and differences between groups evaluated by an unpaired *t*-test. FPG, fasting plasma glucose; T2D, type 2 diabetes.

Fasting plasma glucagon, noradrenaline, somatotropin and cortisol were similar between groups (Fig. 3). During hyperglycemia, plasma glucagon was equally suppressed, although significantly higher in the group of patients with type 2 diabetes compared to controls (Fig. 3A). During hypoglycemia, plasma glucagon, noradrenaline and somatotropin increased in both groups, without any difference in the baseline-subtracted area under the curve. When the hypoglycemic phase was initiated, serum cortisol was already markedly elevated in the group of patients with type 2 diabetes but not in controls (Fig. 3C). Accordingly, the baseline-subtracted area under the curve for serum cortisol was significantly higher in the group of patients with type 2 diabetes.

### Plasma potassium

**Figure 4:**
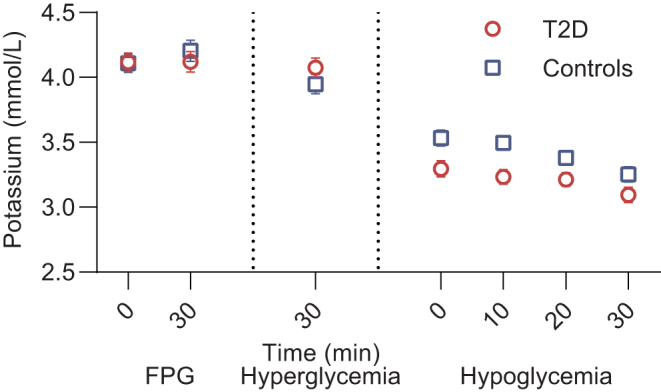
Plasma potassium. Levels of plasma potassium (mean ± s.e., *n*  = 21 in both groups) during each steady-state phase of the experimental day in patients with type 2 diabetes (red circles) and controls (blue squares). FPG, fasting plasma glucose; T2D, type 2 diabetes.

The two groups had similar levels of plasma potassium during the FPG phase (Fig. 4 and Table 2). During hyperglycemia, a significant decrease in plasma potassium was found in controls, whereas it remained unchanged in the group of patients with type 2 diabetes. During the hypoglycemic phase, plasma potassium decreased in both groups compared with the FPG phase with no difference between the groups at the end of the hypoglycemic phase. However, plasma potassium reached a significantly lower nadir during hypoglycemia in the group of patients with type 2 diabetes compared with controls (3.1 mmol/L (2.9, 3.2) and 3.2 mmol/L (3.1, 3.3), respectively, *P* = 0.023).

## Discussion

In the present study, QTc interval increased significantly during insulin-induced acute hypoglycemia in both insulin-treated patients with type 2 diabetes and matched controls with no difference between groups. In a previous study comparing streptozotocin-treated diabetic rats to non-diabetic rats, diabetes was found to aggravate QTc prolongation during hypoglycemia, indicating increased susceptibility to QTc abnormalities in patients with diabetes (15). Similarly, Chow *et al.* found that QTc prolongation during hypoglycemia was greater in patients with type 2 diabetes compared to controls, although the difference was not significant (11). This difference could be explained by longer diabetes duration, the criteria of insulin treatment and a potentially higher incidence of cardiac autonomic neuropathy in the present study (18). The observed increase in QTc interval during hypoglycemia in patients with type 2 diabetes was smaller than what has previously been reported (11, 12, 13) even when applying Bazett’s correction which is known to overcorrect for high heart rates (19). This is likely to reflect the older age and long diabetes duration in the present study, but also a shorter period of hypoglycemia since QTc has been demonstrated to increase progressively for up to 80 min of hypoglycemia (18). Nevertheless, both groups experienced significant maximal QTcB and QTcF interval prolongations above normal range during hypoglycemia and, in half of the individuals in each group, the maximal QTcB interval exceeded 500 ms, which has been identified as an important threshold for risk of syncope, cardiac arrest and sudden cardiac death in patients with the long-QT syndrome (20). Thus, the observed changes should be considered clinically important.

Spontaneous hypoglycemia has previously been demonstrated to increase the incidence of premature ventricular beats in patients with type 2 diabetes (10). In the present study, the incidence of ventricular premature beats increased in both groups during hypoglycemia. Although premature ventricular beats are common in healthy individuals (21), frequent premature ventricular beats have been associated with a substantial increase in the risk of sudden cardiac death (22). The development of atrial fibrillation immediately following hypoglycemia in a single patient in the group of patients with type 2 diabetes adds to a small number of reported cases of hypoglycemia-related atrial fibrillation (23, 24). Although no conclusions can be drawn from these anecdotal cases, frequent hypoglycemia may add to the atrial fibrillation burden in insulin-treated patients with paroxysmal atrial fibrillation. Our findings support recommendations to avoid hypoglycemia in patients with established cardiovascular disease (25).

Both groups had marked counterregulatory responses during hypoglycemia. Interestingly, the cortisol response was significantly higher in patients with type 2 diabetes. High levels of plasma insulin might potentiate cortisol, adrenaline and noradrenaline secretion during hypoglycemia (26, 27) and, in the present study, higher doses of insulin were required to induce hypoglycemia in patients with type 2 diabetes due to pronounced insulin resistance. Hence, it seems likely that the difference in cortisol secretion between the groups, which was evident already when initiating the hypoglycemic phase, may be explained by differences in plasma insulin. Another potential contributing factor could be a higher prevalence of obstructive sleep apnea in the group of patients with type 2 diabetes (sleep apnea is associated with increased cortisol levels (28)). Thus, a limitation of the current study is that we did not include screening for sleep apnea. Nevertheless, there was no difference in cortisol levels during the FPG phase nor the hyperglycemic phase, and the effect of sleep apnea on the hormonal counterregulatory response has not previously been investigated.

The sympathoadrenal response to hypoglycemia is the main mediator of QTc prolongation during hypoglycemia (29). In accordance with previous observations, the response in plasma noradrenaline was similar between patients with type 2 diabetes and controls (11). The threshold for the sympathetic and hormonal counterregulatory response is generally elevated in patients with type 2 diabetes (30, 31), whereas the counterregulatory threshold is normalized when insulin therapy is initiated (32). Furthermore, previous episodes of hypoglycemia attenuate the counterregulatory response during subsequent episodes of hypoglycemia (33). In contrast to previous studies, the present study included only insulin-treated patients with type 2 diabetes with a low HbA_1c_ and, accordingly, a high risk of hypoglycemia. Nevertheless, noradrenaline response to hypoglycemia was unaffected indicating a preserved sympathoadrenal response.

In a previous study in patients with type 2 diabetes and controls, a significant decrease in plasma potassium during hypoglycemia was found only in patients with type 2 diabetes (11). The decline in plasma potassium during insulin-induced hypoglycemia is almost equally mediated by insulin-induced and catecholamine-induced cellular potassium uptake (34). In the present study, there was no significant difference in the decrease in plasma potassium at the end of the hypoglycemic phase between the two groups, whereas plasma potassium nadir was significantly lower in the group of patients with type 2 diabetes. Since no between-group difference in noradrenaline was observed, the difference in plasma potassium nadir is most likely to be explained by the difference in insulin dose between the groups. Since the effect of insulin on PG and plasma potassium is independent (35), insulin-induced cellular potassium uptake is not affected by insulin resistance in type 2 diabetes, which could explain the difference in plasma potassium in spite of almost similar changes in PG. Since hypokalemia is an underlying mechanism behind hypoglycemia-induced abnormal cardiac repolarization (29), large therapeutic doses of insulin to overcome insulin resistance may amplify the degree of hypokalemia during iatrogenic insulin-induced hypoglycemia in type 2 diabetes and, thereby, increase the risk of ventricular arrhythmias. Importantly, even mild hypokalemia is associated with an increased risk of cardiac arrhythmias and death (36, 37).

The present study has some limitations. A rather high insulin dose was applied to induce hypoglycemia within a short period. Although this may not fully resemble most hypoglycemic episodes in real life, cardiac arrhythmia induced by hypoglycemia is likely to be a relatively rare event. In this context, exploring the response to a potent stimulus is relevant. Insulin concentrations were not measured, since the available assay would not detect concentrations of some long-acting insulin analogs in the patients with type 2 diabetes, which would complicate interpretation of data. The difference in time to hypoglycemia and insulin dose between groups complicates the interpretation of between-group differences, although the rate of decline in plasma glucose was almost similar between the two groups. The difference in the dose of insulin required to induce hypoglycemia reflects a relatively high daily insulin dose in these high insulin-resistant patients. The period of hypoglycemia was relatively short mimicking a daytime episode of hypoglycemia, potentially ending before the full effect on cardiac rhythm and repolarization could be observed (18). Lastly, due to over-dispersed data, estimates of the relative incidence of premature beats should be interpreted with caution.

In conclusion, hypoglycemia causes significant and clinically relevant QTc prolongations in patients with type 2 diabetes. The findings add to the evidence supporting a role for hypoglycemia in the induction of cardiac arrhythmias and sudden cardiac death. Noteworthy, any relevant difference in QTc interval prolongation during hypoglycemia between high-risk, insulin-treated patients with type 2 diabetes and controls without diabetes does not appear to exist. Hence, type 2 diabetes itself does not appear to aggravate the repolarization abnormalities caused by hypoglycemia.

## Declaration of interest

A A, M P A B, M B C, K U A, J F, T B L, G G declare that they have no competing interests. J I B has received lecture fee from Novo Nordisk. U P B has served on advisory boards for AstraZeneca/Bristol Myers Squibb, Sanofi Aventis, Novo Nordisk and Zealand Pharma and has received lecture fees and research grants from Novo Nordisk. J J H has served on scientific advisory panels for Novo Nordisk. F K K has served on scientific advisory panels and/or been part of speaker’s bureaus for, served as a consultant to and/or received research support from Amgen, AstraZeneca, Bayer, Boehringer Ingelheim, Carmot Therapeutics, Eli Lilly, Gubra, MedImmune, MSD/Merck, Mundipharma, Norgine, Novo Nordisk, Sanofi and Zealand Pharma. T V has served on scientific advisory panels, been part of speaker’s bureaus for, served as a consultant to and/or received research support from Amgen, AstraZeneca, Boehringer Ingelheim, Eli Lilly, Gilead, Mundipharma, MSD/Merck, Novo Nordisk, Sanofi and Sun Pharmaceuticals.

## Funding

This work was supported by unrestricted grants from the Novo Nordisk http://dx.doi.org/10.13039/501100004191 Foundation (grant number NNF 16230) and the Capital Region of Denmark (grant number E-19280-51-07).

## Availability of data and materials

The datasets generated and analyzed during the current study are not publicly available due to Danish data protection laws but are available from the corresponding author on reasonable request.

## Author contribution statement

A A, J I B, M B C, T B L, G G, F K K and T V designed the study and wrote the protocol. U P B contributed with participant recruitment. A A, M P A B and K U A performed the study. J J H and J F measured glucagon and noradrenaline, respectively. A A performed the data analysis and wrote the first draft. All authors critically revised the manuscript and approved the final version. A A and T V are the guarantors of this work and, as such, had full access to all the data in the study and takes responsibility for the integrity of the data and the accuracy of the data analysis.
